# Crack Detection during Laser Metal Deposition by Infrared Monochrome Pyrometer

**DOI:** 10.3390/ma13245643

**Published:** 2020-12-10

**Authors:** Yin Wu, Bin Cui, Yao Xiao

**Affiliations:** 1 School of Aerospace Engineering, Xi’an Jiaotong University, Xi’an 710049, China; wyxjtu@stu.xjtu.edu.cn; 2 State Key Laboratory for Manufacturing Systems Engineering, Xi’an Jiaotong University, Xi’an 710049, China; dream_joy@stu.xjtu.edu.cn; 3 Collaborative Innovation Center of High-End Manufacturing Equipment, Xi’an Jiaotong University, Xi’an 710049, China

**Keywords:** laser metal deposition, additive manufacturing, crack detection, infrared monochrome pyrometer

## Abstract

Laser metal deposition (LMD) is an advanced technology of additive manufacturing which involves sophisticated processes. However, it is associated with high risks of failure due to the possible generation of cracks and bubbles. If not identified in time, such defects can cause substantial losses. In this paper, real-time monitoring of LMD samples and online detection of cracks by an infrared monochrome pyrometer (IMP) could mitigate this risk. An experimental platform for crack detection in LMD samples was developed, and the identification of four simulated cracks in a 316L austenitic stainless-steel LMD sample was conducted. Data at temperatures higher than 150 °C were collected by an IMP, and the results indicated that crack depth is an important factor affecting the peak temperature. Based on this factor, the locations of cracks in LMD-316L austenitic stainless-steel samples can be determined. The proposed technique can provide real-time detection of cracks through layers of cladding during large-scale manufacturing, which suggests its relevance for optimizing the technological process and parameters, as well as reducing the possibility of cracks in the LMD process.

## 1. Introduction

Laser metal deposition (LMD), also known as laser cladding (LC), laser metal forming (LMF), laser engineered net shaping (LENS), and laser free-form fabrication (LFFF), has been widely used in 3D printing and repair of high value-added metal parts [1,2,3,4,5]. LMD is the most cutting-edge technology in additive manufacturing and rapid prototyping (3D printing) systems, and is an important development direction in the field of intelligent manufacturing [6,7,8,9]. The LMD technology has significant advantages over traditional forming methods, such as casting [10]. By adopting this advanced technology, the manufacturing process becomes relatively simple since no mold is required, and its design and manufacturing are integrated, which, in general, saves substantial quantities of raw materials and processing time [11,12,13]. Meanwhile, LMD is increasingly becoming the research focus in the field of surface modification of modern materials due to its numerous advantages, such as pollution free and possibility to combine different coatings and substrates [14,15,16]. So far, LMD is widely used in mould manufacturing, turbine blade repairing, thermal bamer coatings, and other industrial fields [17,18,19,20].

LMD is a complex technique, which includes melting of metal powders under high temperature followed by immediate cooling. The large temperature gradient may lead to insufficient supplement of liquid metal during the cooling process and contraction due to the relatively cooler adjacent substrate material during the subsequent consolidation process. This can result in stress concentrations, which cannot be released, leading to defects once released [21,22]. Such defects may occur during the manufacturing process and damage the products. Defects such as cracks and pores are highly likely to occur under high temperature and cold air flow environments or due to improper selection of processing parameters during manufacturing [23,24,25]. These defects can seriously weaken the mechanical properties of workpieces and reduce the molding accuracy. Hence, real-time online monitoring during LMD processing is needed to recognize, locate, and tackle defects in time. If such defects are not detected in time, they may lead to disasters, such as in aerospace engineering. Consequently, it is of particular significance and value to develop methods able to effectively detect such defects.

In cladding process of LMD, both macro- and micro-cracks may occur in the cladding layer. The former are normally (hot) cracks often formed near the solidification temperature, and are known as solidification cracks [26]. This type of macro-cracks is usually formed in the solidification temperature range. They result from the insufficient supplement of liquid metal when the consolidation and contraction processes suddenly release stress after increased thermal stress issued from the rapid cooling process [27,28]. Micro-cracks are mainly related to slip zone cracking, phase interface cracking, and grain boundary cracking. They are usually produced by uneven local slip and micro-cracking of metal materials. The uneven slip of a material is generally formed only on the material surface. As the slip band continues to widen, when a certain level is reached, micro-cracks are generated at the location where the slip band resides. Meanwhile, micro-cracks can also be found in small cavities, impurities, and uneven crystal grains generated during material processing [29,30].

Non-destructive testing methods have been widely applied in various fields, such as aerospace, petrochemical, nuclear industry, and machinery manufacturing [31,32,33,34]. Radiographic testing is a defect detection method which utilizes rays, such as gamma rays, neutron rays, and X-rays, to pass through dense materials, such as metal components, in order to determine, based on the degree of ray attenuation, whether there are cracks, pores, and other defects [35,36,37,38,39]. Nevertheless, this detection method is harmful to the human body [40,41], as well as expensive and inapplicable to on-line monitoring. On the other hand, metal magnetic powder inspection can locate defects on welded parts by measuring the variations in the magnetic field caused by the faulty parts [42,43,44]. However, this method makes the manufacturing process more difficult due to the induced magnetic field. Acoustic emission (AE) signal processing is a dynamic nondestructive testing method, which can accurately determine the nature and size of defects when there is certainty signal [45,46,47,48,49]. The signal curve characteristics can be used to determine whether there is damage or its degree in the internal structure of a workpiece. During LMD processing, the temperature of the sample can reach up to hundreds of degrees centigrade, and the AE sensor is in close contact with the surface of the test pieces via silica gel. The temperature of the silica gel should be lower than 100 °C, which limits to a certain degree the application of AE detection [50,51]. Moreover, the most difficult problems of the AE signal processing method are the diversity of sources, bursts, and uncertainty of signals [52,53,54,55]. A large number of uncertain signals from the laser-molten metal also increase the difficulty of detecting defect signals in AE signal processing. The infrared detection technology can continuously monitor the thermal state of the test pieces through a non-contact infrared system, and, at the same time, diagnose whether the pieces have defects based on subtle changes in temperature [56,57].

In a word, the radiation produced by radiographic testing can cause irreversible harm to humans and machines. Magnetic particle testing lays particular emphasis on the smoothness of the test part surface. This method sets high requirements on the employed techniques and experience of the inspectors. Moreover, it is also devalued by its small detection range and slow detection speed. Acoustic emission detection cannot be applied to such experiments due to the external interference from the LMD. Compared with the aforementioned methods, infrared detection stands out with its simple operation, high sensitivity, high temperature resolution and extremely short response time. It is usually used for non-contact online temperature measurement during rapid heating. When the surface temperature of the defective LMD test piece between 385 and 450 °C, the temperature change can be identified when its surface is scanned by the IGAR 12-LO high-precision infrared two-color pyrometer. The researchers in Ref. [58] noticed this phenomenon, but failed to offer theoretical explanation for its cause; thus, this observation could not be used to justify the temperature change corresponding to the defect. The measurement range of the IGAR 12-LO high-precision infrared two-color pyrometer is 350–1300 °C, which meant that it cannot used on larger-sized LMD samples, when their surface temperature is below 350 °C. In addition, the two-color thermometer was expensive, which increases the LMD processing cost after failure.

Thus far, only a handful of papers dealing with the detection of defects during LMD have been published. In this paper, simulated cracks on the surface of a LMD sample are accurately identified by an IMP. The crack is detected accurately by the temperature curve acquired by the IMP. The operating temperature of the IMP ranged between 150 °C and 1200 °C. The proposed method can monitor the crack generation, crack size, and track the position of the crack in real-time during the LMD process. The cracks could be removed in time through the subtractive manufacturing method. When no defects were detected by the IMP, the LMD process was continued. The detection method presented in this paper is simple, convenient, inexpensive, and practical.

## 2. Crack Detection Principles

### 2.1. Crack Detection Principles Using an Infrared Monochrome Pyrometer

**Figure 1:**
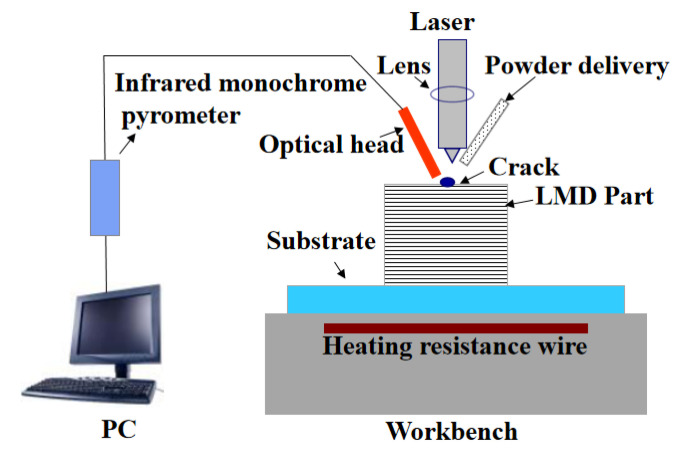
Schematic diagram of crack detector configuration using the IMP.

In metal cladding, after the metal powder was melted by the laser, an ultra-high temperature environment was formed in the molten pool. Under the cold air flow, the molten metal solidified instantaneously, and a large temperature difference was developed on the surface of the molten pool. This large temperature difference introduced large thermal stress. At the same time, it also affected the microstructure of the cladding layer, causing microscopic crystals to deviate from the processing direction and grow disorderly. This disorder gave rise to a greater structural stress. The thermal and structural stresses could not be offset, and macroscopic defects, such as cracks, appeared. Figure 1 shows a schematic diagram of the LMD crack detection configuration. A dismountable substrate was fixed on the workbench, while metal powder melts were exposed to the laser beam to form the product on the substrate following a layer-by-layer process. The optical head of the infrared monochrome pyrometer (IMP; Model IGA320/23-LO IMPAC^®^ Pyrometer, LumaSense Corporation, Frankfurt, Germany) was mounted on one side of the LMD nozzle. The IMP would collect temperature information at each point of the LMD specimen surface. The IMP optical head has a built-in LED target light, and the extremely small spot size was convenient for aiming at the object to be tested. Moreover, the parts to be tested could be accurately located based on the position of the red spot. The operating temperature of the IMP ranged between 150 and 1200 °C, and the size of the focal spot was 0.45 mm. A heating resistance wire was installed under the worktable as auxiliary heating for the substrate, and a temperature sensor was used to control the upper and lower temperature limits of the substrate.

**Figure 2:**
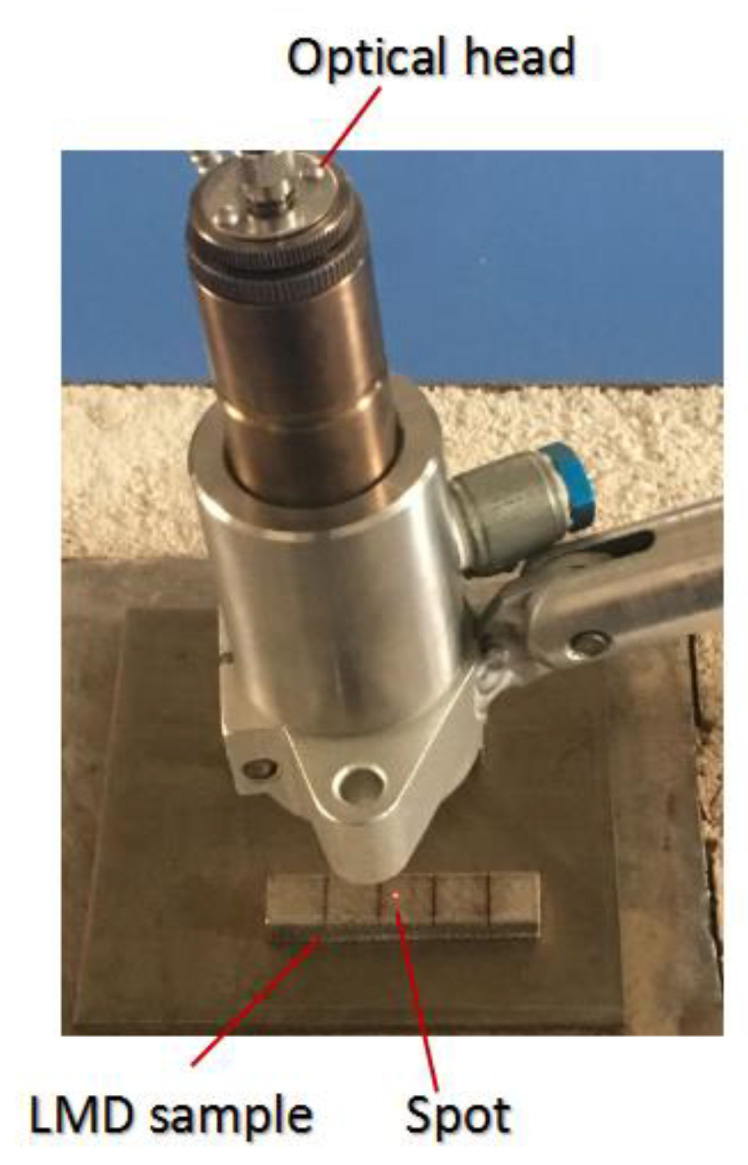
LMD specimen surface temperature collection by the IMP.

Figure 2 depicts the temperature acquisition method on the LMD specimen surface using a non-contact IMP with an emissivity of (0.1, 1), hysteresis of 2 °C, and transmission 100%. The red spot in Figure 2 was used to locate the position of the tested specimen.

**Figure 3:**
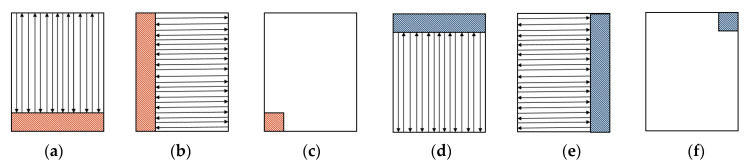
Scanning paths of the LMD nozzle. (**a**) Scanning path of the first layer; (**b**) scanning path of the second layer; (**c**) after scanning the first and the second layers, the part indicated by the square at the lower left corner is not scanned by the IMP; (**d**) scanning path of the third layer; (**e**) scanning path of the forth layer; (**f**) after scanning the third and the fourth layers, the part indicated by the square at the upper left corner is not scanned by the IMP.

In real LMD 3D Printing [59], the IMP optical head was fixed on the LMD nozzle by a rotatable fixture, which could adjust the relative position of the nozzle and IMP optical head. The direction of the arrow in Figure 3a,b,d,e represents the movement direction of the LMD nozzle.

The specific method is as follows:

❖ Step 1: In the first-layer, the LMD nozzle is directly behind the IMP, and the shaded part in Figure 3a is not scanned.
❖ Step 2: In the second-layer, the LMD nozzle scans perpendicular to the scanning direction of the previous layer. The fixture rotates 90° clockwise. The LMD nozzle is still directly behind the IMP optical head; thus, the shaded part of Figure 3b is not scanned. After scanning the two layers, only the small square at the lower left corner of Figure 3c has not been scanned.
❖ Step 3: During the production of the third layer, the LMD nozzle returns to the original position of the first layer, and the fixture continues to rotate 90° clockwise so that the IMP is in front of the nozzle. After the third layer has been completed, it can be seen that the small square at the lower left corner has been completely covered. When scanning the third layer, the shadow part of Figure 3d is not scanned by the IMP.
❖ Step 4: The fixture continues to rotate 90° clockwise, and the fourth layer is formed. The IMP is still in front of the LMD nozzle, scanning perpendicular to the scanning direction of the previous layer. The shaded part of Figure 3e is not scanned. Therefore, after scanning the third and the fourth layers, only the small square at the upper left corner of Figure 3f has not been scanned. The fixture continues to rotate 90° clockwise, and it can be seen that the nozzle has returned to the initial state when the first layer was made, and the IPM is directly behind the LMD nozzle.
❖ Step 5: The first four steps are repeated until the part processed by LMD is completed. The scanning of the entire area by the IMP can be realized by the sequential scanning of the four layers.

A computer was used to analyze the scanned data and evaluate the quality of the LMD part. When cracks appear in the cladding layer, they can be accurately located through the IMP cracks detection method and be removed in time through the subtractive manufacturing mechanism. After all cracks have been completely removed, the processing can continue by cyclically repeating the first for steps.

### 2.2. Plane Wall Heat Conduction

To detect the temperature change of the upper surface of the LMD test piece by IMP, it is necessary to develop a heat transfer model.

The Fourier law [60] is given by Equation (1):

(1) $$q=\frac{\Phi }{A}$$

The heat transfer rate [61] is given by Equation (2):

(2) $$\Phi =-\lambda \times A\times \frac{dT}{d\delta }$$

In Equations (1) and (2), where *A* is the isothermal surface area (m^2^), *q* is the heat flux (W/m^2^), Φ represents the heat transfer rate (W). Concerning the temperature gradient, temperature differences exist between isothermal surfaces with different temperatures and the ratio of temperature difference to vertical distance difference. *dT/dδ* is the temperature gradient, Φ indicates that the heat flow direction is always opposite to that of the temperature gradient, and T is heat transfer temperature value.

The LMD model is regarded as a stack of several planes, and the heat from the heat source at the bottom of the substrate is transferred layer-by-layer to the upper surface through each plane. In the modeling process, the following assumptions were made [62,63]:

(i) The thermal conductivity λ is constant (only one nozzle).
(ii) The heat loss from the plane wall edge can be ignored.
(iii) The temperature of the plane wall varies only along the vertical wall surface direction rather than time.

**Figure 4:**
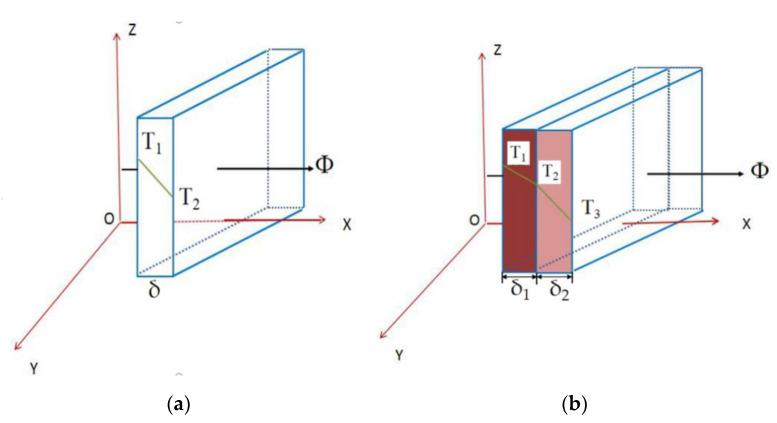
Schematic diagram of layer conduction of the plane wall. (**a**) Single-layer; (**b**) Multi-layer.

Figure 4a illustrates a schematic diagram of single-layer conduction and Figure 4b a schematic diagram of multi-layer conduction. During the process of temperature conduction through the layer-by-layer deposition process, heat accumulation or loss can occur in uneven structures. The temperature changes were collected in real-time by the IMP, and defects were identified and analyzed.

- Single-layer conduction (Figure 4a)

Firstly, a single-layer plane heat transfer model is established.

If *T*_1_ > *T*_2_, then

(3) $$\Phi =-\lambda \frac{A(T_{2}-T_{1})}{\delta },\delta =x_{2}-x_{1},\delta >0$$

(4) $$\frac{\Phi }{A}=-\frac{T_{2}-T_{1}}{\frac{\delta }{\lambda }},\;\mathrm{or}\;T_{2}-T_{1}=-\frac{\delta }{\lambda }\times \frac{\Phi }{A}$$

Note that: *δ* denotes the distance parallel to the heat flow direction, (*T*_1_ − *T*_2_) is the heat loss value after a heat transfer displacement *δ*, *x*_1_ and *x*_2_ are spatial coordinates, δ = (*x*_2_ − *x*_1_) is the heat transfer displacement, *A* is the isothermal surface area, *λ* is the thermal conductivity, and Φ represents the heat transfer rate.

- Multi-layer conduction (Figure 4b)

LMD is formed through stack of multi-layers, and each layer is similarly regarded as a conductive plane. LMD detects the temperature change of the uppermost layer. Thus, this is a multi-layer heat conduction problem.

For the first layer, $\delta_{1}=x_{2}-x_{1}$, $T_{1}>T_{2}$:

(5) $$T_{2}-T_{1}=-\frac{\delta_{1}}{\lambda_{1}}\times \frac{\Phi_{1}}{A_{1}}$$

For the second layer, $\delta_{2}=x_{3}-x_{2}$, $T_{2}>T_{3}$:

(6) $$T_{3}-T_{2}=-\frac{\delta_{2}}{\lambda_{2}}\times \frac{\Phi_{2}}{A_{2}}$$

For the nth layer, $\delta_{n}=x_{n+1}-x_{n}$, $T_{n}\;>T_{n+1}$:

(7) $$T_{n+1}-T_{n}=-\frac{\delta_{n}}{\lambda_{n}}\times \frac{\Phi_{n}}{A_{n}},n\in N^{+}$$

Note that: *δ_n_* (*n* ∈ *N*^+^) denotes the distance parallel to the heat flow direction, (*T_n_* − *T_n_*_+1_) is the heat loss value after heat transfer displacement *δ_n_*, *x*_*n*_ and *x*_*n*+1_ are spatial coordinates, *δ*_i_ = (*x_n_*_+1_ – *x_n_*) is the heat transfer displacement, *A*_i_ is the isothermal surface area, λ*_n_* is the thermal conductivity, and Φ*_n_* represents the heat transfer rate.

## 3. Experimental Methods

### 3.1. Experimental Set-up

**Figure 5:**
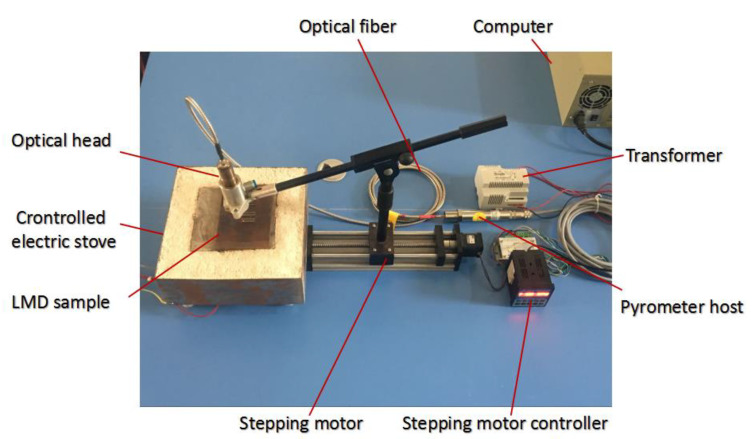
The experimental configuration used to detect the cracks in LMD samples.

Figure 5 presents the device platform used for detecting defects in LMD samples. An LMD sample was placed on the temperature-controlled furnace substrate. The bottom of the furnace was heated by a resistance wire and temperature was set between 175 to 230 °C. Using optical cables, one side of the IMP host was connected to the optical head and the other to an industrial controlling machine. The IMP host collected the data in real-time through a dedicated software.

**Figure 6:**
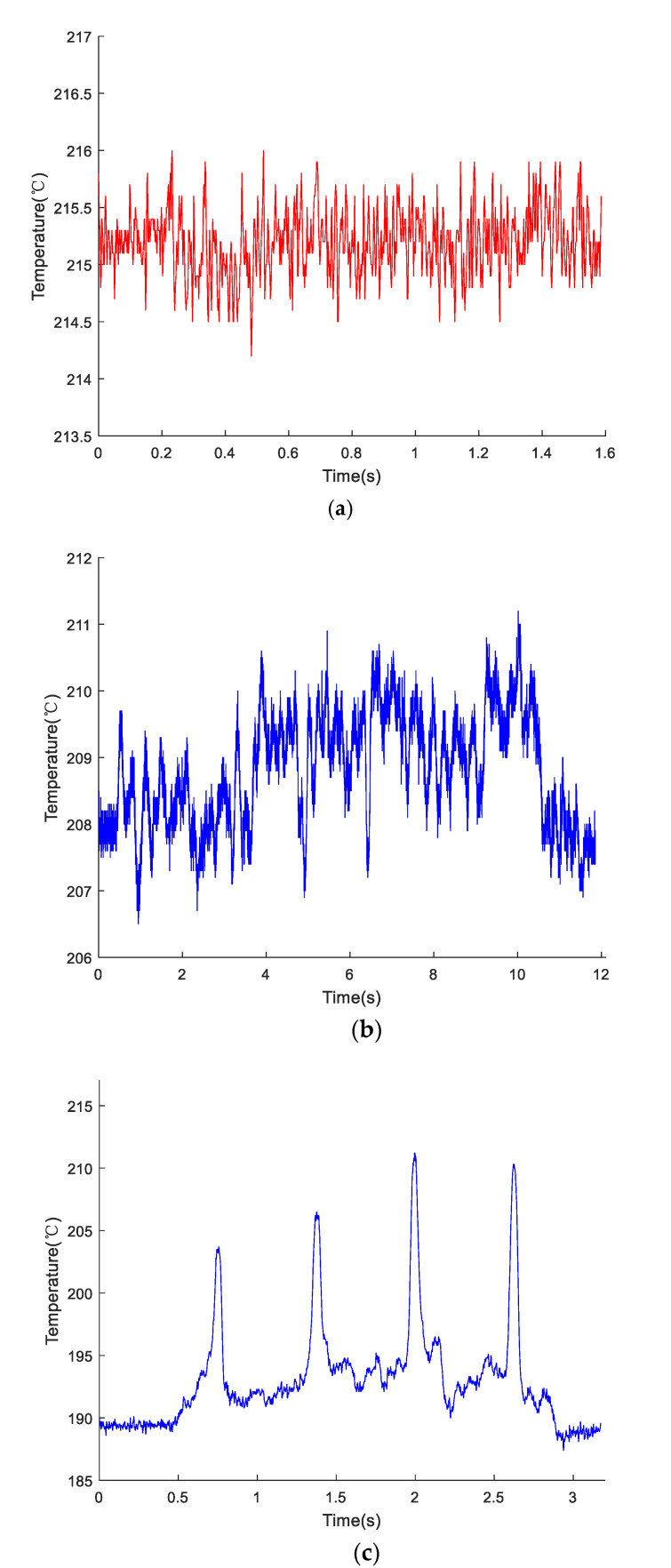
Collected temperature data using the IMP. (**a**) Temperature changes of the upper surface of the furnace; (**b**) Temperature changes of the upper surface of the non-defective LMD sample; (**c**) Surface temperature changes with time of a defective LMD sample.

The motion speed and direction of the optical head were controlled by a stepping motor controller. The optical head was adjusted on the stepping motor to scan the sample upper surface. During scanning, the temperature at different positions of the LMD sample upper surface was collected in real-time. The quality of the sample could be determined based on the changes in temperature. Figure 6 presents the acquired temperature data using the IMP. It can be seen that the change in temperature was related to the surface roughness and other parameters. 

The temperature values of the optical head navigating on the upper surface of the furnace for 1.6 s were recorded by the IMP (Figure 6a). Little changes in the temperature can be observed, and the maximum temperature difference was 1.5 °C. The non-defective LMD sample was placed on the upper surface of the furnace, and the focus spot of the optical head navigated on the upper surface for 12 s (Figure 6b). The temperature variation on the upper LMD sample surface was recorded by the IMP. It can be seen that the temperature range was not large, the maximum temperature difference was 5 °C, and no obvious or special peak values were found. When the optical head navigated on the LMD sample with simulated defects for 3 s, four apparent peaks in the temperature curve were observed, and the maximum temperature difference was close to 30 °C (Figure 6c). It can be deduced that the IMP has high sensitivity.

### 3.2. Crack Simulation Experiments

**Figure 7:**
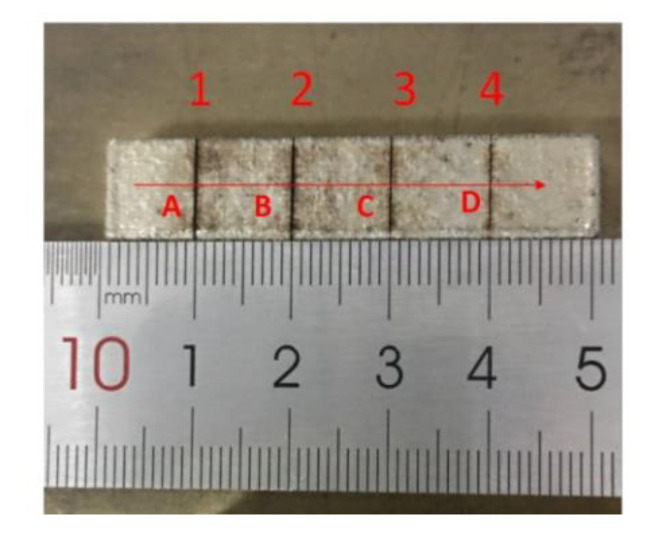
316L austenitic stainless-steel sample made by LMD.

Atomized 316L stainless-steel powder supplied by BGRIMM Advanced Materials Science & Technology Co., Ltd. (Beijing, China), was used as the material to produce the LMD samples. Its nominal chemical composition is reported in Table 1. Figure 7 shows a sample made of 316L austenitic stainless-steel powder by LMD. The size of the sample was 50 mm × 10 mm × 7 mm (length × width × height), and four crack defects with different widths and depths were introduced on the sample surface by a laser.

**Figure 8:**
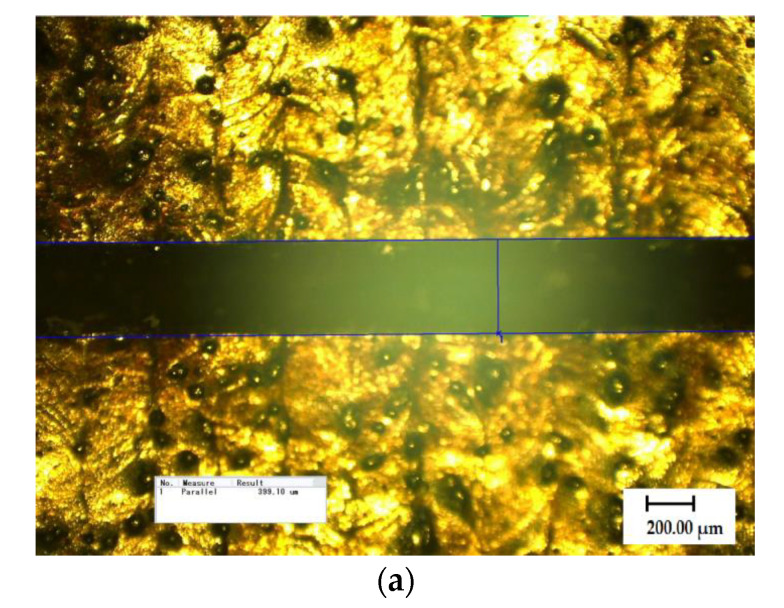

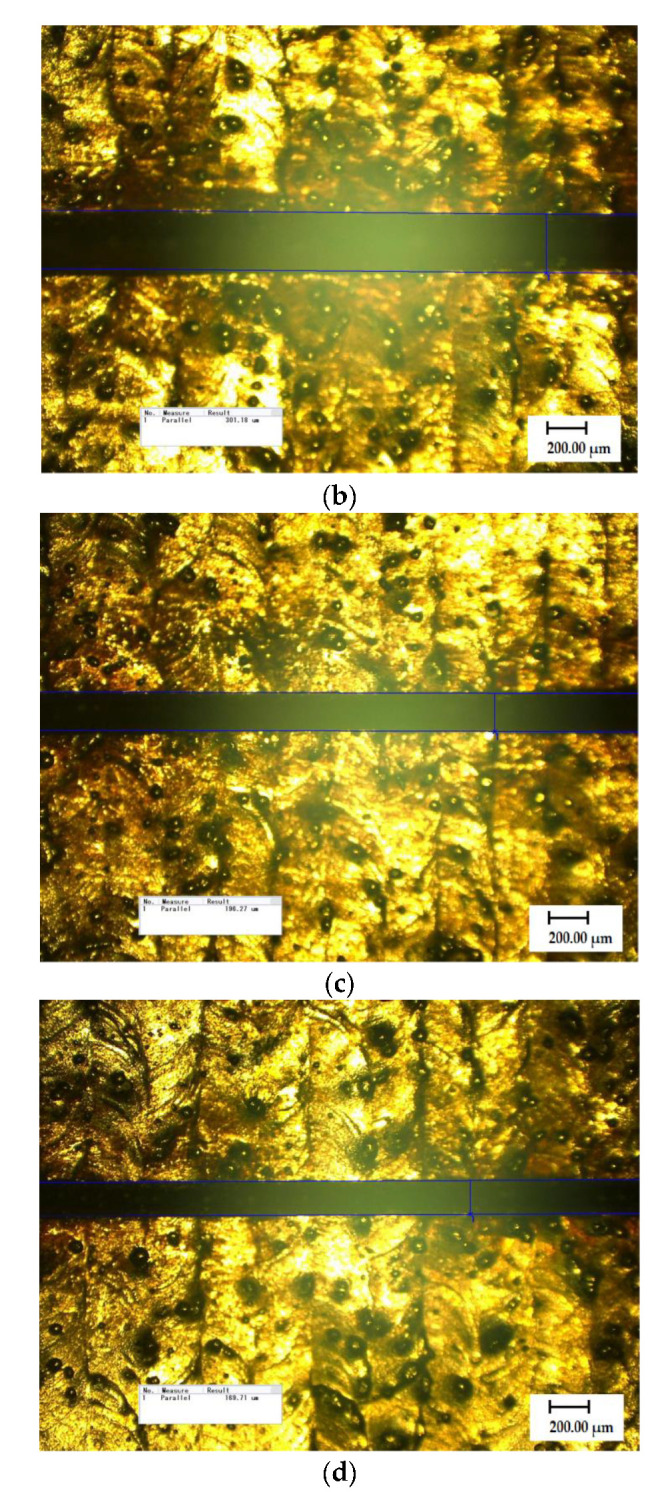
Simulated crack defects on an LMD-316L austenitic stainless-steel sample observed under optical microscopy (×100): (**a**) Crack 1; (**b**) Crack 2; (**c**) Crack 3; and (**d**) Crack 4.

Figure 8a–d present the structure diagram of the sample around the four simulated crack defects under optical microscopy (KEYENCE VH-8000, Keyence microscopes, Tokyo, Japan). The surface of the sample appeared scraggly and some slags were coated with unclad metal. The width of each defect was measured by optical microscopy. The depth of each defect was measured under laser scanning confocal microscope (LSCM, LEXT 3D measuring laser microscope OLS4000, Olympus corporation, Tokyo, Japan).

It should be noted that after starting the experiment, the stepping motor drove the optical head to the perpendicular to the crack (1–4). The direction and speed of the optical head were controlled by a stepping motor controller. In order to facilitate presentation, the direction of the red arrow shows the positive direction of the optical head motion, and the opposite direction of the red arrow shows the opposite direction of the optical head motion. Thus, the rule was that the “1→2→3→4” direction was the positive direction, and that of 4→3→2→1 was set as the opposite direction (Figure 7).

In Table 2, the dimensions of the simulated crack defects are given. The width and depth of Crack 1 were measured to be 399.10 μm and 208.23 μm, respectively. The width and depth of Crack 2 were measured as 301.18 μm and 280.13 μm, respectively. The width and depth of Crack 3 were 196.27 μm and 165.10 μm, respectively. The width and depth of Crack 4 were measured to be 169.71 μm and 122.71 μm, respectively.

**Figure 9:**
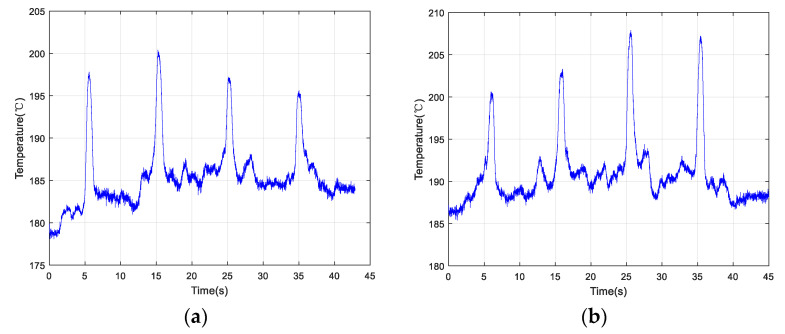
Collected temperature data of the four cracks scanned by an optical head at 1.0 mm/s along the (**a**) positive direction and (**b**) opposite direction.

Figure 9a shows an example of the scan along the positive direction. At a scanning speed of 1.0 mm/s, the changing order of the peak value estimated based on the temperature change was 2→1→3→4 (ranking from largest to smallest). Figure 9b depicts another scan example along the opposite direction at the same speed. The order of the peak temperature determined from the temperature curve was 2→1→3→4 (ranking from largest to smallest).

**Figure 10:**
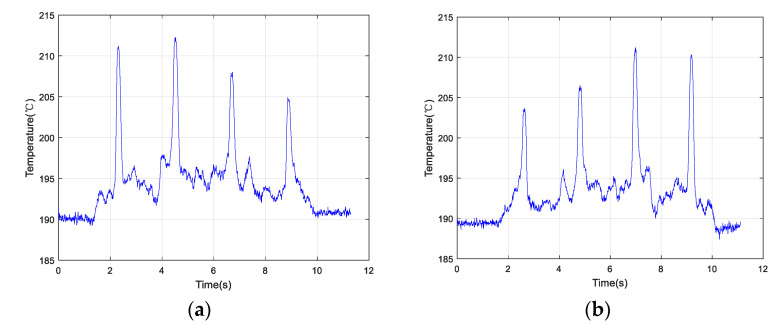
Collected temperature data of the four cracks scanned by an optical head at 5.0 mm/s along the (**a**) positive direction and (**b**) opposite direction.

**Figure 11:**
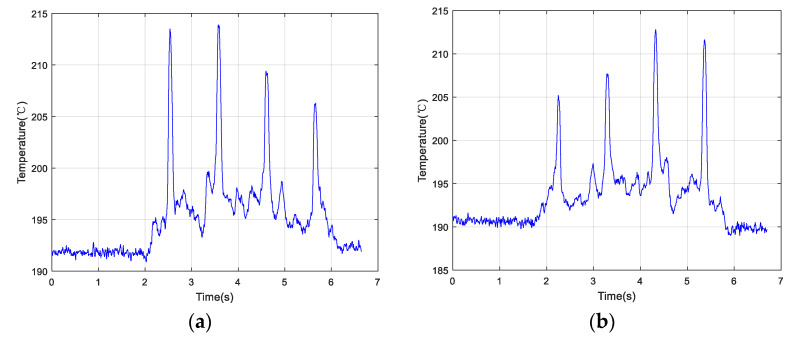
Collected temperature data of the four cracks scanned by the optical head at 10.0 mm/s along the (**a**) positive direction and (**b**) opposite direction.

In order to reduce occasionality, two groups of experiments were conducted by changing the scanning speeds. Figure 10a demonstrates the change in temperature as a function of time along the positive direction at 5.0 mm/s. Figure 11b shows the temperature change as a function of time along the opposite direction at 5.0 mm/s. The order of peak temperature change was recorded as 2→1→3→4 and the changing trends of the two plots appeared very similar.

The temperature data collected along the positive and negative directions at a speed of 10.0 mm/s are shown in Figure 11a,b, respectively. Using three sets of experimental data, the temperature change curves exhibited the same shapes when the optical head passed over the simulated crack defects. The order of peak temperature values according to both plots was 2→1→3→4. Therefore, the quality of the LMD sample surface could be inspected by the IMP by analyzing the temperature change curve, indicating that the proposed method is feasible.

In Table 3, the variation of the peak temperature values under different scanning speeds is given. At the same speed, the temperature value of Crack 2 determined from the curve was always maximal, while that of Crack 4 was minimal.

## 4. Results and Discussion

At floor heating mode, the IMP conducted an online detection of simulated cracks on the LMD specimen surface. The heat flow was transmitted in a layer-upon-layer fashion. The transmission to uneven surfaces would accumulate and diffuse heat. The IMP recorded the temperature changes.

### 4.1. Confirmation Cracks

The direction of red arrow in Figure 7 indicates the positive scanning direction (“1→2→3→4”), which was perpendicular to the crack slot. The intersection of the vertical crack slot of the motion trajectory was marked as A, B, C, D. This means that |AB| was the distance between Crack 1 and Crack 2, |BC| the distance between Crack 2 and Crack 3, and |CD| the distance between Crack 3 and Crack 4. It is easy to obtain that |AB| = |BC| = |CD| = 10 mm by measuring with a scale.

**Figure 12:**
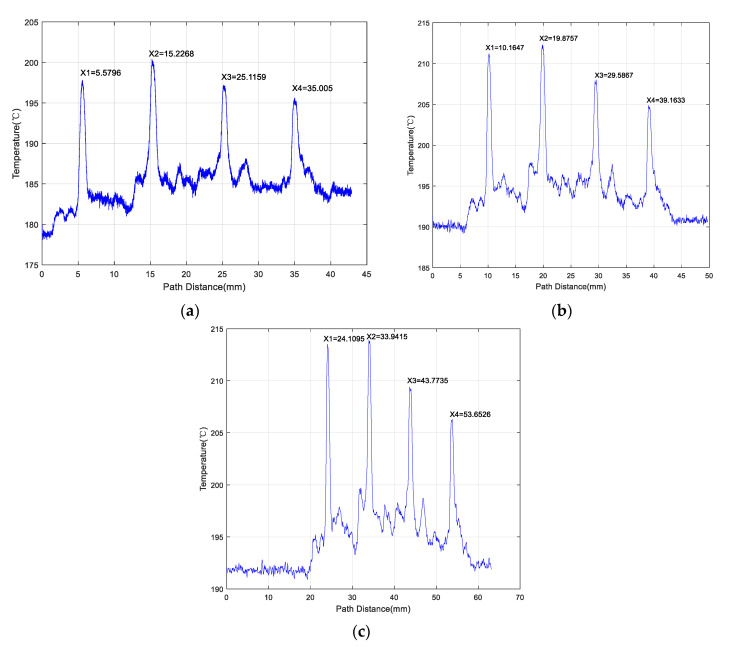
Temperature waveforms obtained along the positive direction of the LMD-316L austenitic stainless-steel sample upper surface at a scanning speed of (**a**) 1.0 mm/s; (**b**) 5.0 mm/s; and (**c**) 10.0 mm/s.

Figure 12 indicates the positions of the optical head at the different temperature peaks. In Cartesian coordinates, the horizontal axis represented the position of the optical head, and the vertical axis the temperature. X1, X2, X3, and X4 are the points of the four curve peaks (horizontal coordinate) as the optical head moved along the positive direction (1→2→3→4) (Figure 7). Furthermore, |X2 − X1|, |X3 − X2|, and |X4 − X3| are the distances between adjacent peaks.

In Table 4, it is easy to find that the values of |X2 − X1|, |X3 − X2|, and |X4 − X3| were all close to 10 mm. In other words, |X2 − X1| ≈ |X3 − X2| ≈ |X4 − X3|.

Consequently, it was determined that the peaks in the temperature change curve coincided with the positions of the specimen cracks. The changes in the temperature curve can correct the expression of the specimen surface roughness. Hence, the proposed method used to detect cracks on LMD samples using an IMP is valid and effective.

### 4.2. Cracks Influencing Factors

**Figure 13:**
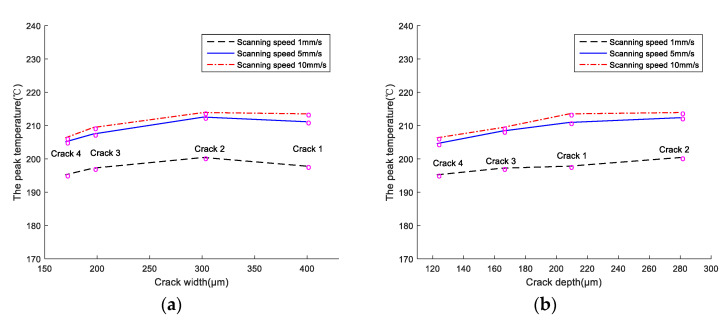
Curves of peak temperature versus crack (**a**) width and (**b**) depth obtained from the LMD-316L austenitic stainless-steel workpiece with simulated cracks.

As it can be seen in Figure 13a, the shape of the peak temperature versus crack width curves under different speeds (1.0 mm/s, 5.0 mm/s, and 10.0 mm/s) was similar. The curves revealed an obvious increasing trend for crack widths ranging between 160 and 300 μm, and a decreasing trend for crack widths larger than 300 μm. Figure 13b indicated a proportional relationship between crack depth and peak temperature. The deeper the crack depth, the higher the peak temperature.

According to the test results, the IMP was able to detect cracks on metal surfaces. At different scanning speeds, obvious temperature changes were observed at the crack locations. The upward or downward trends of the curves were similar. In addition, for the same crack length, the peak temperature values were related to the width and depth of the cracks.

A theoretical explanation of the numerical heat transfer theory using the Fourier law is as follows.

According to Equation (7):

(8) $$(T_{2}-T_{1})+(T_{3}-T_{2})+\ldots +(T_{n+1}-T_{n})=-(\frac{\Phi_{1}}{A_{1}}\times \frac{\delta_{1}}{\lambda_{1}}+\frac{\Phi_{2}}{A_{2}}\times \frac{\delta_{2}}{\lambda_{2}}+\ldots +\frac{\Phi_{n}}{A_{n}}\times \frac{\delta_{n}}{\lambda_{n}}),n\in N^{+}$$

(9) $$T_{n+1}-T_{1}=-(\frac{\Phi_{1}}{A_{1}}\times \frac{\delta_{1}}{\lambda_{1}}+\frac{\Phi_{2}}{A_{2}}\times \frac{\delta_{2}}{\lambda_{2}}+\ldots +\frac{\Phi_{n}}{A_{n}}\times \frac{\delta_{n}}{\lambda_{n}}),n\in N^{+}$$

Equations (8) and (9) are equivalent, Equation (9) is a simplified form of Equation (8). In general, the initial value *T*_1_ of the heating process would be the same, and the value of temperature *T_n_*_+1_ to be determined would depend on Φ, *A*, *λ*, and *δ*.

**Figure 14:**
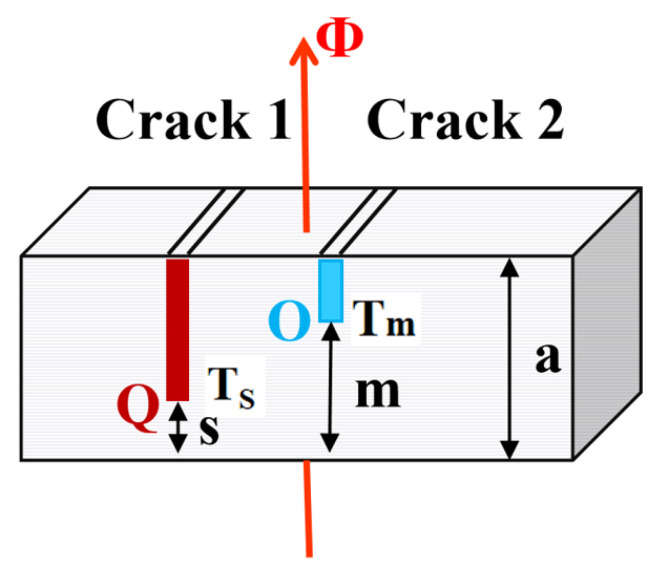
Dimensions of cracks on the LMD specimen.

❖ According to Equation (9), if Φ, *A*, *λ*, and *δ* are the same, the fluctuation of the curve would be small, and *T_n_*_+1_ would be on the same plane (isothermal surface) even if there are no cracks on the LMD workpiece.
❖ If the height of the LMD specimen (Figure 14) was a, the distance between point Q and the substrate would be s in Crack 1, and the distance between point O and the substrate would be m in Crack 2. The depth of Crack 1 would be *h_s_* = (a − s) and that of Crack 2 would be *h_m_* = (a − m). In the heat transfer process of the LMD-316L austenitic stainless-steel cuboid sample, the values of Φ and *λ* would be similar.

(10) $$T_{n+1}-T_{1}=-(\frac{\Phi_{1}}{A_{2}}\times \frac{\delta_{1}}{\lambda_{1}}+\frac{\Phi_{2}}{A_{2}}\times \frac{\delta_{2}}{\lambda_{2}}+\ldots +\frac{\Phi_{n}}{A_{n}}\times \frac{\delta_{n}}{\lambda_{n}})=-\frac{\Phi }{\lambda }\times (\frac{\delta_{1}}{A_{1}}+\frac{\delta_{2}}{A_{2}}+\ldots +\frac{\delta_{n}}{A_{n}}),n\in N^{+}$$

- Q point:

(11) $$T_{s}-T_{1}=-\frac{\Phi }{\lambda }\times (\frac{\delta_{1}}{A}+\frac{\delta_{2}}{A}+\ldots +\frac{\delta_{s}}{A})=-\frac{\Phi }{\lambda }\times \frac{s}{A}=-\frac{\Phi }{\lambda }\times \frac{\left(a-h_{s}\right)}{A}=-\frac{\Phi a}{\lambda A}+\frac{\Phi h_{s}}{\lambda A}$$

(12) $$T_{s}=-\frac{\Phi a}{\lambda A}+\frac{\Phi h_{s}}{\lambda A}+T_{1}$$

- O point:

(13) $$T_{m}-T_{1}=-\frac{\Phi }{\lambda }(\frac{\delta_{1}}{A}+\frac{\delta_{2}}{A}+\ldots +\frac{\delta_{m}}{A})=-\frac{\Phi }{\lambda }\frac{m}{A}=-\frac{\Phi }{\lambda }\times \frac{\left(a-h_{m}\right)}{A}=-\frac{\Phi a}{\lambda A}+\frac{\Phi h_{m}}{\lambda A}$$

(14) $$T_{m}=-\frac{\Phi a}{\lambda A}+\frac{\Phi h_{m}}{\lambda A}+T_{1}$$

(15) $$T_{s}-T_{m}=\left(-\frac{\Phi a}{\lambda A}+\frac{\Phi h_{s}}{\lambda A}+T_{1}\right)-\left(-\frac{\Phi a}{\lambda A}+\frac{\Phi h_{m}}{\lambda A}+T_{1}\right)=\frac{\Phi h_{s}}{\lambda A}-\frac{\Phi h_{m}}{\lambda A}=\frac{\Phi }{\lambda A}(h_{s}-h_{m})$$

When the heat transfer rate in the test piece remains unchanged, if *h_s_* > *h_m_*, then *T_s_* > *T_m_*. This means that the deeper the crack depth, the greater the temperature value recorded by the IMP. This is explained in Table 2, which shows the depth values of the LMD-316L austenitic stainless-steel simulated cracks, indicating that the sequence of the crack depth values (2→1→3→4) was the same as that of peak temperature values (2→1→3→4).

## 5. Conclusions

In summary, the IMP was able to detect cracks on the LMD sample based on the variations in temperature due to accumulation and loss of heat when the infrared radiation reached uneven structures. The proposed real-time online crack detection method was found to be feasible, simple, and reliable. The following conclusions can be drawn:

1. The evaluation of surface cracks on a 316L austenitic stainless-steel sample prepared by LMD using an IMP indicated that the absence of cracks on the specimen surface induced smaller temperature changes with stable trends. In other words, if there are cracks on the LMD sample surface, the temperature changes at the crack would be significant. Remarkably, it was found that the temperature peaks in the plots were located at the exact same position with the defects.
2. When the heat transfer rate in the test piece remained unchanged, the IMP was able to rapidly detect the cracks and determine their sizes. In addition, it was able to scan the cracks at different speeds and various directions. For curves with similar trend and stability, the up or down trends in the surface temperature curves of the LMD sample were unrelated to the optical head scanning speed. The changes in the temperature curve were related only to the crack depth of the LMD workpiece. Deeper depths induced larger temperature changes.
3. The combination of additive manufacturing and subtractive manufacturing can guarantee the high quality of LMD samples. When defects appear in the cladding layer, they can be accurately located using the proposed real-time online defect detection method and be removed in time through the subtractive manufacturing mechanism. After all defects have been completely removed, the processing can continue through the additive manufacturing mechanism.

## Figures and Tables

**Table 1:** Chemical composition of the 316L austenitic stainless-steel powder.

Composition	Content (wt.%)
C	≤0.030
Mn	≤2.00
S	≤0.01
Si	0.030
P	0.045
Cr	17.5–18
Ni	12.00
Mo	3.00
Fe	Bal.

**Table 2:** The size of the simulated cracks on the LMD-316L austenitic stainless-steel sample.

Crack No	Width (μm)	Depth (μm)
Crack 1	399.10	208.23
Crack 2	301.18	280.13
Crack 3	196.27	165.10
Crack 4	169.71	122.71

**Table 3:** Peak temperature variation for the different cracks at different scanning speeds (statistical positive data).

Crack No	Scanning Speed		
	1.0 mm/s	5.0 mm/s	10.0 mm/s
	Peak Temperature (°C)		
Crack 1	197.8	211.2	213.5
Crack 2	200.4	212.3	213.9
Crack 3	197.2	208.0	209.4
Crack 4	195.2	204.7	206.3

**Table 4:** Distance between adjacent peaks in the temperature-distance plots obtained under different speeds.

Distance between Peaks (mm)	Scanning Speed		
	1.0 mm/s	5.0 mm/s	10.0 mm/s
|X2 − X1|	9.6472	9.7110	9.8320
|X3 − X2|	9.8891	9.7110	9.8320
|X4 − X3|	9.8891	9.5766	9.8791
